# Impact of pre-analytic step duration on molecular diagnosis of toxoplasmosis for five types of biological samples

**DOI:** 10.1371/journal.pone.0246802

**Published:** 2021-02-17

**Authors:** Marie-Pierre Brenier-Pinchart, Emmanuelle Varlet-Marie, Florence Robert-Gangneux, Denis Filisetti, Juliette Guitard, Yvon Sterkers, Hélène Yera, Hervé Pelloux, Patrick Bastien

**Affiliations:** 1 Laboratoire de Parasitologie-Mycologie, CHU Grenoble Alpes et Institut pour l’Avancée des Biosciences (IAB), INSERM U1209-CNRS UMR 5309, Université Grenoble Alpes Grenoble, Grenoble, France; 2 Centre National de Référence Toxoplasmose-Pôle Biologie Moléculaire, France; 3 Université de Montpellier et Laboratoire de Parasitologie-Mycologie CHU Montpellier, Montpellier, France; 4 CHU Rennes, Inserm, EHESP, Irset (Institut de Recherche en Santé Environnement Travail), UMR_S 1085, Université de Rennes, Rennes, France; 5 Institut de Parasitologie et de Pathologie Tropicale, Université de Strasbourg et Laboratoire de Parasitologie et Mycologie Médicale, Hôpitaux Universitaires de Strasbourg, Strasbourg, France; 6 Inserm, Centre de Recherche Saint-Antoine, CRSA, AP-HP, Hôpital Saint-Antoine, Sorbonne Université, Paris, France; 7 CNRS, IRD, CHU de Montpellier, "MiVEGEC" et Laboratoire de Parasitologie-Mycologie, Université de Montpellier, Montpellier, France; 8 Laboratoire de Parasitologie-Mycologie, Hôpital Cochin, Université de Paris, AP-HP, Paris, France; Golestan University of Medical Sciences and Health Services, ISLAMIC REPUBLIC OF IRAN

## Abstract

**Introduction:**

*Toxoplasma*-PCR is essential to diagnose ocular, cerebral, disseminated and congenital toxoplasmosis. This multicenter study evaluated the impact of sample storage duration at +4°C on PCR assay performances in order to propose guidelines for the storage of samples during shipment or/and before PCR.

**Materials and methods:**

Five matrices, amniotic (AF), cerebrospinal (CSF), and bronchoalveolar lavage fluids (BALF), whole blood (WB) and buffy coat (BC), were artificially spiked with different amounts of *Toxoplasma gondii* (20, 100, 500 tachyzoites per mL of sample) or with previously infected THP1 cells. DNA extractions were performed at day 0 and after 2, 4 and 7 days of storage at +4°C. Each extract was amplified at least twice by real-time PCR.

**Results:**

A total of 252 spiked samples was studied. No increase of crossing point was observed and all samples were positive for AF, BALF, BC and infected THP1-spiked WB after up to 7 days at 4°C. For CSF spiked with 20 parasites/mL, only 50% of PCR reactions were positive at D7 (p<0.05). For WB spiked with type II parasites, all reactions remained positive at D7 but amplifications were significantly delayed from D2; and for WB spiked with RH strain, the proportion of positive reactions decreased at D7.

**Conclusion:**

The storage of clinical samples at +4°C is compatible with the molecular detection of *T*. *gondii* parasites. Provided that PCR assays are performed in duplicate, storage of samples is possible up to 7 days. However, from the fifth day onwards, and for samples susceptible to contain low parasitic loads, we recommend to perform the PCR in multiplicate.

## Introduction

Toxoplasmosis is a widespread zoonosis caused by *Toxoplasma gondii*, an effective obligate intracellular protozoan parasite [1]. Transmission to humans results from the ingestion of oocysts shed by infected felids and of cysts from undercooked meat of infected animals. It is generally assumed that approximately 25 to 30% of the world’s human population is infected by *Toxoplasma* with large disparities across the world [1, 2]. In immunocompetent individuals, primary infection is mostly asymptomatic or accompanied by mild, nonspecific and self-limited signs. However, two main subpopulations are highly susceptible to this parasite: the fetus and the immunocompromised individual. Congenital toxoplasmosis (CT) occurs in infants following maternal infection. CT may result in fetal death and abortion and in syndromes that include neurologic and neurocognitive deficits and chorioretinitis. The global annual incidence of CT was estimated to be 190,100 cases worldwide [3]. Immunocompromised patients (HIV, hematopoietic stem cell transplant (HSCT), solid organ transplant (SOT) patients, or patients with other immune deficiency) are at risk for life-threatening opportunistic forms of toxoplasmosis as a consequence of several physiopathological mechanisms [4]. The estimation of the incidence of toxoplasmosis in allo-HSCT and SOT patients ranges from 0% to 16% and 0.08% to 25%, respectively [5]. Lastly, ocular toxoxplasmosis occurs in immunocompromised and immunocompetent patients: toxoplasmic retinochoroiditis is the most common form of posterior uveitis in many countries, particularly in South America [6]. Molecular detection of *T*. *gondii* today plays a crucial role in the diagnosis of congenital, ocular, cerebral, pulmonary and disseminated toxoplasmosis. *Toxoplasma*-PCR is performed on various biological samples collected from patients according to the clinical form of toxoplasmosis, e.g. amniotic fluid (AF), aqueous humor, cerebrospinal (CSF) or bronchoalveolar lavage (BALF) fluids and blood [7–11]. It is still very often based upon ’laboratory-developed’ PCR assays, leading to great variation of diagnostic performances among laboratories [12]. Still, an optimal sensitivity of PCR is required because parasitic loads are often low in these samples.

Multiplex molecular panels are currently being developed to diagnose respiratory tract, bloodstream and meningitis/encephalitis infections [13]. Even though these tests can be performed on some of the samples used to diagnose toxoplasmosis, none of these panels includes the detection of *Toxoplasma* DNA. In the absence of a syndrome-based approach, the molecular diagnosis of toxoplasmosis still relies on a targeted diagnostic approach performed in proficient laboratories.

In this context, it is of paramount importance to optimize all steps of this specific molecular diagnosis, including the pre-analytical step [14–17]. As samples may travel for more than 24 hours before arriving in proficient laboratories, it is important to evaluate the impact on the result of the delay between sampling and PCR implementation. Moreover, this assessment is a part of the laboratory Quality Management System, all the more so since the diagnosis of congenital toxoplasmosis may have consequences in terms of civil and medical liability. Furthermore, this problematic is not restricted to the molecular diagnosis but also concerns the storage of sera [18].

Here, we examined this issue in a multicenter study, by using seven types of artificially spiked biological samples stored at +4°C. We assessed the impact of sample storage duration on *Toxoplasma*-PCR performances after 2, 4, and 7 days at +4°C, with a view to proposing guidelines for the shipment and the delay before DNA extraction is performed.

## Materials and methods

The six participating laboratories are proficient in detecting *T*. *gondii* in clinical specimens; they are members of the "Molecular biology" group of the French National Reference Centre for Toxoplasmosis and participate to the external quality assessment (EQA) for *Toxoplasma*-PCR [19, 20]. Moreover, four of them hold an agreement from the Ministry of Health (Regional Health Agency) for realizing the prenatal diagnosis of toxoplasmosis.

### Mimic samples preparations

Five biological matrices were studied: AF, CSF, BALF, whole blood (WB) and buffy coat (BC). AF, CSF and BALF samples were stored at -20°C, and WB and BC samples at + 4°C, before the addition of *T*. *gondii* [14, 15]. The volume of artificially spiked samples was adapted to be close to that of the different matrices in routine practice: 2 mL of AF, 200 μL of CSF, 500 μL of BALF, and 1 mL and 4 mL of WB (hereafter termed “small WB” and “large WB”, respectively). The BC samples were provided by the French Blood Centre (Etablissement Français du Sang) and 160 μL of BC, corresponding to approximately 2.5 mL of blood, were used. The above-mentioned matrix volumes were used for each concentration/sample; and each type of artificial sample was made up and tested in one centre.

To make up artificial samples, parasites of the RH strain, the Prugniaud (PRU) strain and a type II strain isolated from a patient, were harvested from *in vitro* cultivated human foreskin or MRC5 fibroblasts and counted using a Malassez cell. In each centre, a stock suspension of 5×10^4^ free tachyzoites/mL was prepared in phosphate-buffered saline (PBS) and immediately used to spike samples at the final concentrations of 500, 100 and 20 *T*. *gondii*/mL, except for BALF which was spiked at 1000, 200 and 40 *T*. *gondii*/mL [14, 15]. In addition, with the objectives of examining the fate of samples spiked with intracellular *T*. *gondi*, whole blood was also spiked with a human monocyte cell line (THP-1, ATCC® TIB202) previously infected with the Type II patient’s strain. Briefly, THP-1 were cultured in RPMI 1640 medium (Gibco) supplemented with 10% fetal calf serum, 100 IU/mL penicillin, and 100 μg/mL streptomycin, and infected with type II parasites (MOI 5:1). Three days post-infection, the cell suspension was collected and the number of cells was counted using a Malassez cell. It was then deposited on cytospin slides by cytocentrifugation; and after Giemsa staining, the percentage of infected cells was determined under the microscope, which allowed inferring the number of infected cells. Four mL of WB were then spiked with 500, 100 and 20 infected THP-1 cells/mL (“Large WB”). The preparation of samples is summarized in Table 1. All spiked samples were made in triplicate for each parasite concentration, and then stored at +4°C.

**Table 1:** Details of the pre-analytic and analytic steps implemented for the different *T*. *gondii*-spiked samples.

Sample	*T*. *gondii* strain	Starting concentration in samples Tg/mL	Sample volume spiked and stored at +4°C before DNA extraction (Tg/mL)	Pre-processing just before extraction	Sample volume extracted	DNA extraction protocol	Elution volume (number of Tg^1^/mL)	PCR Apparatus	Volume of DNA extract/PCR reaction	Calculated number of Tg/ PCR reaction^2^
AF	RH	500/100/20	2000 μL (1000/200/40)	No	2000 μL	TNN^3^	50 μL (1000/200/40)	In-house PCR LightCycler 480	5 μL	100/20/4
CSF	RH	500/100/20	200μL (100/20/4)	No	200 μL	QIAamp DNA Mini Kit (Qiagen)	200 μL (100/20/4)	In-house PCR LightCycler 480	6.5 μL	3.25/0.65/0.13
BALF	RH	1000/200/40	500 μL (500/100/20)	Pellet of centrifugation	200 μL	QIAamp DNA Mini Kit (Qiagen)	50 μL (500/100/20)	Bioevolution (Stratagene MX3005)	5 μL	50/10/2
WB	Prugniaud	500/100/20	1000 μL (500/100/20)	Pellet of Centrifugation after serial cells lysis	100 μL	MagNA Pure 96 DNA and Viral NA Small Volume Kit (Roche)	100 μL	In-house PCR LightCycler 2.0	5 μL	25/5/1
WB	RH	500/100/20	4000 μL (2000/400/80)	Centrifugation Buffy coat collection	200 μL	QIAamp DNA Mini Kit (Qiagen)	100 μL (2000/400/80)	In-house PCRStepOne Plus	5 μL	100/20/4
WB	Infected THP1 with type II	500/100/20 infected THP-1 cells	(2000/400/80 infected THP-1 cells)				100 μL			Not calculable
BC	RH	500/100/20	160 μL (2.5 mL of blood) (1250/250/50)	No	160 μL	Proteinase-K, boiled and protein precipitation^4^	60 μL (1250/250/50)	In-house PCR LightCycler 480	5 μL	100/20/4

AF: amniotic fluid; CSF: cerebrospinal fluid; BALF: bronchoalveolar lavage fluid; WB: whole blood; BC: buffy coat; Tg: *Toxoplasma gondii*.

^1^ Number of Tg in total volume of elution was calculated with the hypothesis that the DNA extraction performance was 100%.

^2^ Calculated number of Tg/PCR tube = number of Tg in total volume of elution x volume of DNA extract used in PCR tube/elution volume.

^3^ TNN: Tween-Nonidet-NaOH (0.5% Tween 20, 0.5% Nonidet P40, 10 mM NaOH) lysis buffer method [23].

^4^ DNA extraction technique described in Sterkers Y *et al*., 2012 [24].

### DNA extractions and PCR amplifications

Sample processing and DNA extractions were performed at day 0 and after 2, 4 and 7 days of storage at +4°C using methods used in routine in each centre and published previously [12, 16, 20, 21]. Pre-analytical and analytical details are described in Table 1. Briefly, the whole of the volumes of AF, CSF and BC samples were used for DNA extraction; for BALF, 200 μL of pellet obtained after centrifugation (20 000 g, 10 min) of 500 μL were extracted. For the ’small WB’ sample spiked with the PRU strain (small WB+PRU), 1 mL of whole blood was lysed twice with 10 nM Tris-HCl pH 7.5, 0.32 M Sucrose, 5 mM MgCl_2_, 1% Triton X100, and centrifuged at 4400 g for 3 min; 100 μL of pellet were submitted to an external cell lysis with MagNA Pure 96 Bacterial Lysis Buffer with proteinase K (Roche, Meylan, France) according to the manufacturer’s recommendations before DNA extraction. Finally, the 4 mL of ’large WB’ (both WB+RH and WB+infected THP-1) were centrifuged at 1500 g for 15 min, and BCs were collected for DNA extraction [22].

DNA extraction methods varied among laboratories (Table 1): essentially, one manual method included the Tween-Nonidet-NaOH (TNN; 0.5%Tween 20, 0.5% Nonidet P40, 10 mM NaOH) lysis buffer method [23] and a treatment by Proteinase-K (56°C, 12 hours), followed by boiling at 100°C for 10 min and protein precipitation (Protein precipitation A7951, Promega, Charbonnières, France) [24]; commercial kits, used according to the manufacturer’s specifications, were the QIAamp DNA Mini Kit (Qiagen, Courtaboeuf, France) and the MagNA Pure 96 DNA and Viral NA Small Volume Kit (Roche Molecular Biochemicals, Meylan, France]). All DNA extracts were frozen at −20°C until PCR was carried out [17]. Each DNA extract was then amplified in duplicate using real-time PCR assays. All laboratories performed real-time PCR targeting the ‘rep529’ non-coding DNA element (GenBank accession number AF487550) [12, 25, 26]: five laboratories used their own laboratory-developed PCR assay and one used a commercialized *Toxoplasma*-PCR kit (Bio-Evolution, Bussy-Saint-Martin, France). The laboratory-developed PCR assays were performed using a LightCycler 2.0, LightCycler 480 (Roche, Meylan, France) or StepOne (ThermoFisher Scientific, Montigny-le-Bretonneux, France) apparatus. PCR amplification with the Bio-Evolution kit was performed as recommended by the manufacturer on a Stratagene MX3005 (ThermoFisher Scientific). All six real-time PCR methods were thoroughly optimized and were previously assessed as highly performing methods [13, 20, 27].

The results, for each sample at each parasite concentration, were expressed like previously, as a ’PCR performance score’, corresponding to the number of positive amplifications over the total number of PCR reactions performed [12], and as a mean of the crossing point values ± standard deviation (Cp ± SD). Results were analyzed using Fisher’s exact and Wilcoxon rank tests; a p value of 0.05 or less was considered to be significant.

### Ethical approval and informed consent

The different matrices were obtained from the participating centres respecting the Quality Assurance scheme and legal policies. This work was carried out in accordance with the relevant French guidelines and regulations; it does not include potentially identifying patient/participant information. The study corresponds to a non-interventional retrospective study and according to the French Health Public Law (CSP Art L1121-1.1), such studies are exempt from informed consent requirement and do not require approval by an ethics committee. By contrast, in accordance with the French regulations, written consent was obtained before any AF sampling.

## Results

In total, five matrices and seven types of spiked samples were examined; 252 samples were DNA-extracted, yielding 648 PCR reactions. All matrices were controlled and found to be *Toxoplasma*-PCR negative before adding the parasites. All data are reported in S1 Table.

**Fig 1:**
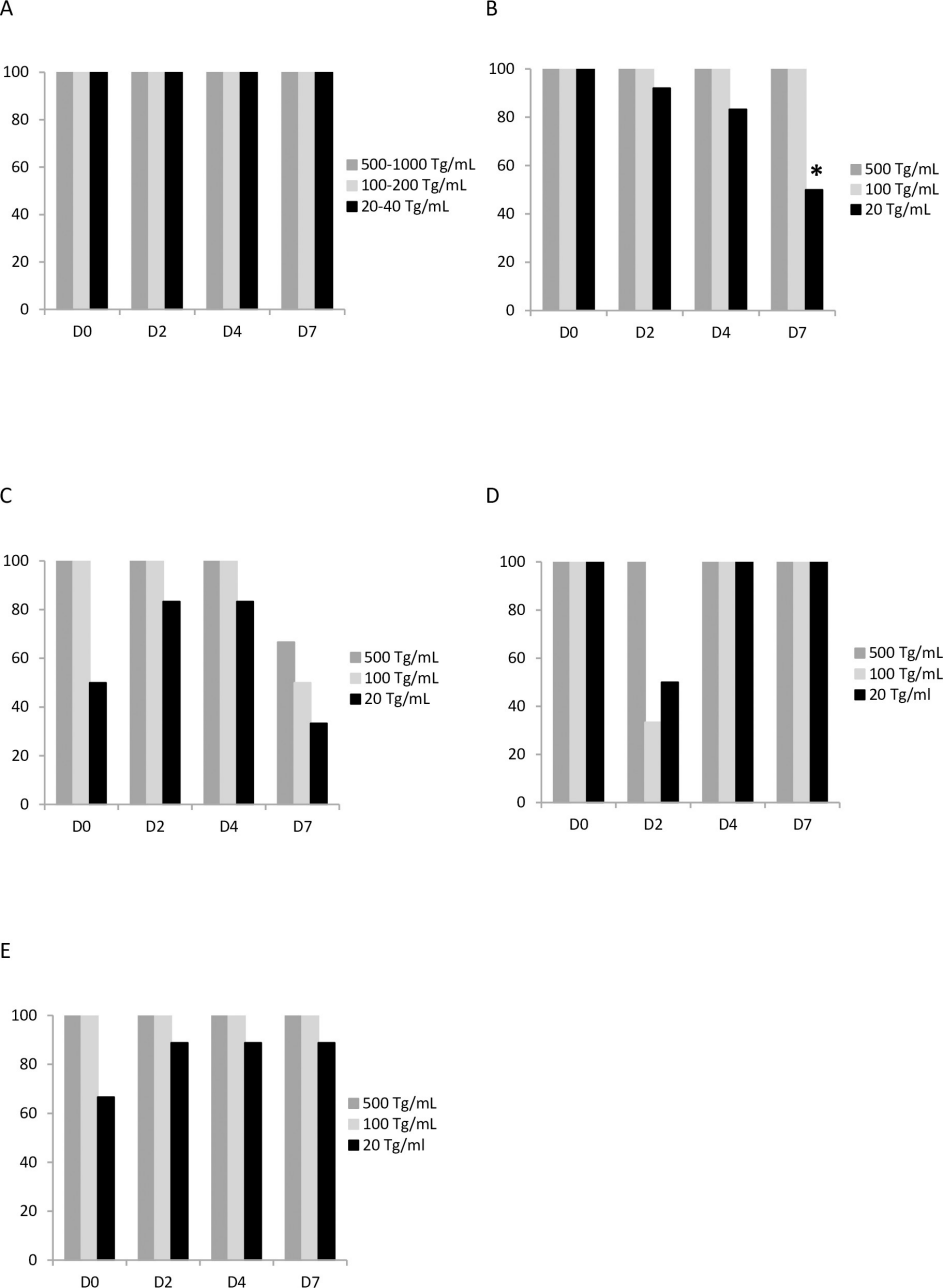
PCR performance scores observed for different samples and parasite concentrations (per mL). A: AF, BAL and ’small WB + PRU’; B: CSF; C: ’large WB + RH’; D: ’large WB + infected-THP1’; E: BC. AF: amniotic fluid; BALF: bronchoalveolar lavage fluid; WB: whole blood; CSF: cerebrospinal fluid; BC: buffy coat; Tg: *Toxoplasma gondii;* RH: RH *Toxoplasma* strain; PRU: Prugnaud *Toxoplasma* strain. *p< 0.05 compared to PCR performance scores without conservation (D0).

**Fig 2:**
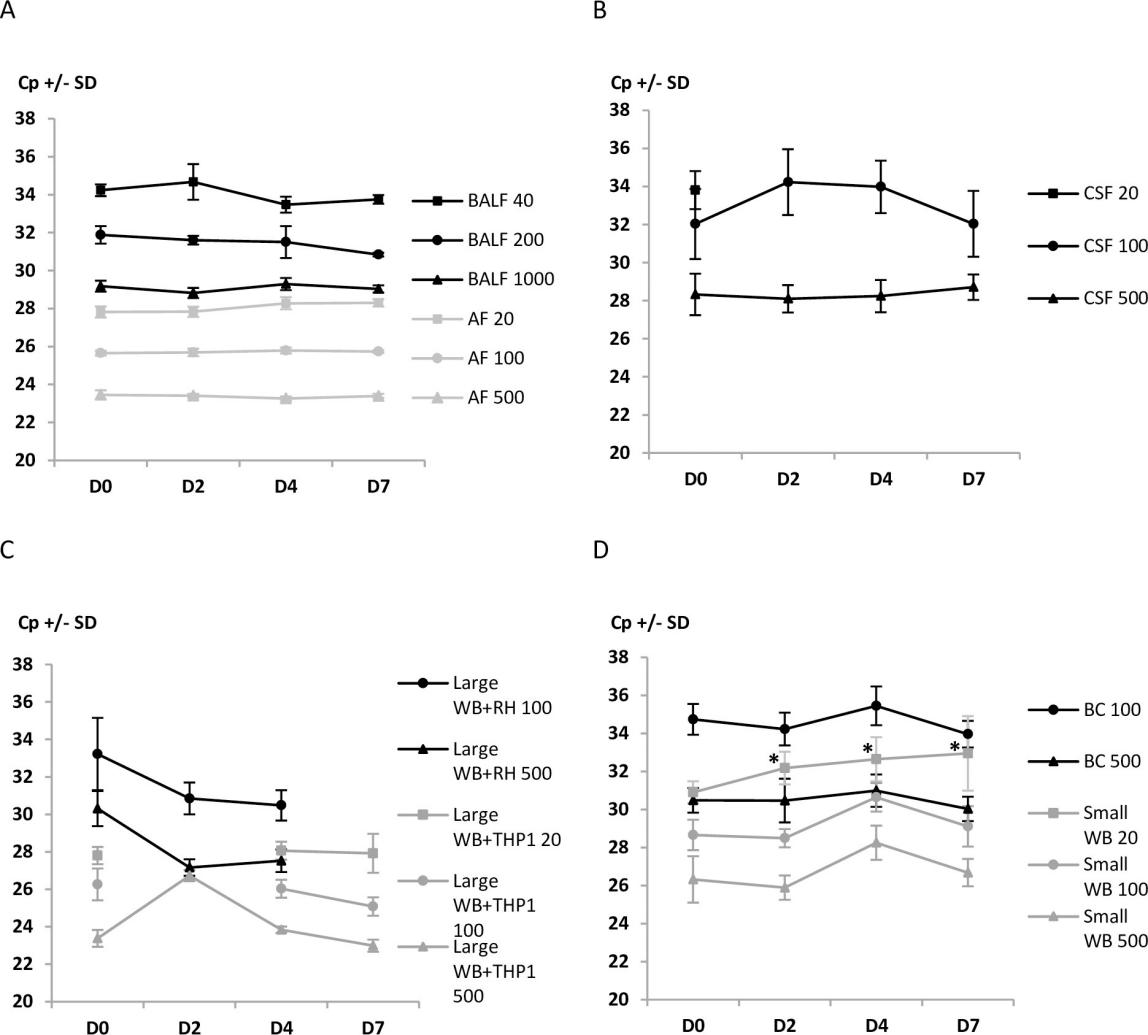
Crossing point values of PCR performed on seven different *T*. *gondii*-spiked samples without and after storage at +4°C during 2, 4 and 7 days before processing for molecular diagnosis of toxoplasmosis. A: BALF and AF; B: CSF; C: ’large WB + RH’ and ’large WB + infected-THP1’; D: BC and ’small WB + PRU’. For each storage duration at + 4°C and *T*. *gondii* concentration (Tg/mL), three different samples were extracted in parallel and at least 2 PCR were performed for each sample. Cp means ± SD were reported only if the PCR performance score was 100%. Tg: *Toxoplasma gondii*; Cp: crossing point; SD: standard deviation; AF: amniotic fluid; CSF: cerebrospinal fluid; BALF: bronchoalveolar lavage fluid; WB: whole blood; BC: buffy coat; RH: RH *Toxoplasma* strain. * p<0.05 compared with Cp measured in samples without conservation.

In paucicellular samples, for AF and BALF, 100% of the PCR reactions were positive and no increase in Cp was observed over storage duration, at all concentrations tested (Fig 1A). Thus, at the lowest *Toxoplasma* concentration, the means of Cp were 27.82±0.6 and 28.30±0.25 at D0 and D7, respectively, after storage at +4°C, for AF; and 34.23±0.31 and 33.75±0.23, respectively, for BALF (Fig 2A). For the CSF with the lowest *T*. *gondii* concentration, the proportion of positive PCR reactions significantly decreased with time and only 50% of reactions were positive at D7 (p<0.05) (Fig 1B, Table 2).

**Table 2:** Details of PCR performance scores and mean of Cp values for a selection of five *T*. *gondii*-spiked samples without and after storage at +4°C during 2, 4 and 7 days before processing for molecular diagnosis of toxoplasmosis (for complete results, see S1 Table).

Sample type (No. of samples extracted)	Inoculum Tg/mL		Without storage (D0)	After 2 days (D2)	After 4 days (D4)	After 7 days (D7)
CSF + RH	20	PCR+ ^a^	12/12	11/12	10/12	6/12*
(n = 37)		Cp ±SD ^b^	33.81±2.06	[34.50±1.42]	[34.50±1.77]	[33.69±1.77]
Small WB + PRU	20	PCR+	9/9	9/9	9/9	9/9
(n = 37)		Cp ±SD	30.90±0.58	32.18±0.86*	32.64±1.16*	32.95±1.96*
Large WB +RH,	500	PCR+	6/6	6/6	6/6	4/6 (NS)
and BC after storage		Cp ±SD	30.31±0.94	27.16±0.44	27.52±0.60	[33.65±5.09]
(n = 37)	100	PCR+	6/6	6/6	6/6	3/6 (NS)
		Cp ±SD	33.22±1.93	30.85±0.85	30.48±0.81	[34.84±3.28]
	20	PCR+	3/6	5/6	5/6	2/6
		Cp ±SD	[34.58±2.86]	[33.93±0.97]	[33.37±2.66]	[35.79±3.52]
Large WB +	100	PCR+	6/6	2/6^c^	6/6	6/6
infected-THP-1and		Cp ±SD	26.26±0.85	[30.10±1.59]	26.03±0.48	25.08±0.49
BC after storage	20	PCR+	6/6	3/6^c^	6/6	6/6
(n = 37)		Cp ±SD	27.80±0.46	[35.41±4.40]	28.06±0.48	27.92±1.04
BC + RH	20	PCR+	6/9	8/9	8/9	8/9
(n = 37)		Cp±SD	[36.68±0.76]	[34.95±1.92]	[36.56±2.05]	[35.38±1.12]

For each storage duration and each *T*. *gondii* concentration, three different samples were extracted in parallel and at least 2 PCR were performed for each sample.

Tg: *Toxoplasma gondii*; Cp: crossing point; SD: standard deviation; AF: amniotic fluid; CSF: cerebrospinal fluid; WB: whole blood; BC: buffy coat; RH: RH *Toxoplasma* strain; PRU: Prugniaud *Toxoplasma* strain; NS: number of positive PCR at D7 not statistically significant compared to number of positive PCR without conservation (D0).

* p<0.05 compared with Cp measured in samples without conservation.

^a^: number of positive PCR reactions/ number of reactions performed.

^b^: mean of Cp ±SD.

^c^: a technical problem during the buffy coat isolation may explain these two discrepant data (see the Discussion section).

For ’small WB + PRU’, PCR performance scores were 100% whichever the parasite concentration tested (Fig 1A). However, at 20 Tg/mL, the Cp mean, which was 30.90±-0.58 before storage (D0), significantly increased with storage duration: 32.18±0.86 at D2, 32.64±1.16 at D4 and 32.95±1.96 at D7 (p<0.05) (Fig 2D, Table 2). This increase was not observed with the two highest parasite concentrations.

For ’large WB + RH’, samples spiked at the lowest *Toxoplasma* concentration were not always positive whatever the storage duration; and the number of positive reactions decreased for the three concentrations at D7 of storage (Fig 1C, Table 2), however this decrease was not statistically significant. No impact of storage duration was observed on ’large WB + infected-THP1’ samples, regardless of the *T*. *gondii* concentration (Figs 1D and 2C).

For the last template, *i*.*e*. BC collected from 2.5 mL of whole blood, storage did not alter parasite detection at intermediate and high parasite concentrations; performance scores were lower for the lowest concentration whichever the storage duration (Figs 1E and 2D).

## Discussion

The biological diagnosis of toxoplasmosis today relies on nucleic acid amplification methods; and because parasitic loads are often low in human samples [28], optimal sensitivity is required in these PCR assays. Technical recommendations (e.g. DNA extraction, *Toxoplasma*-PCR…) and good laboratory practices have already been proposed by the French National Reference Centre for Toxoplasmosis [19]. We also evaluated the impact of long-term storage of *T*. *gondii* DNA extracted from AF samples at -20°C and found that these samples were reliable for retrospective molecular analyses [17]. However, the pre-analytical steps (shipment duration and storage temperature) are also major factors influencing technical performances, and they need to be assessed in order to provide recommendations aiming at warranting steady PCR performances in proficient laboratories. Hence, we explored the impact of the duration of sample storage at +4°C for several types of biological samples routinely collected to diagnose toxoplasmosis in various clinical settings.

Our study shows that *T*. *gondii* parasites appear to be robust in human samples. Indeed, until 7 days after storage at +4°C, provided that PCR was performed at least in duplicate for each sample, the majority of spiked sample types (AF, BALF, ’small WB+PRU’, WB+infected-THP1 and BC) were positively detected for the three parasite concentrations tested. This was different for CSF and ’large WB+RH’ samples. For CSF, the PCR performances significantly decreased only at the 7^th^ day of storage and for very low parasitic loads. Regarding ’large WB+RH’, a strong defect in parasite detection at the lowest concentration was observed at D0. Because this defect was not observed later with an amplification being more efficient after storage for 2 and 4 days, this defect was likely explained by a problem in the preparation of this spiked sample (Table 2). A decrease in PCR performances was visible on D7. It is noteworthy that WB spiked with type II-infected THP1 overall yielded better results than WB spiked directly with RH tachyzoites. However, while the use of intracellular type II parasites is closer to the natural *Toxoplasma* infection, the standardization of the parasite amounts in infected cells is far more difficult, and it is likely that final parasite concentrations were higher in ’large WB+THP1’ than in ’large WB+RH’. In addition, we observed that THP1 cells present in whole blood sometimes make the collection of buffy-coat more difficult. This phenomenon could explain the weak PCR performance score observed 2 days after conservation, whereas these scores were better and homogeneous after 5 and 7 days of conservation.

In this study, we endeavored to preserve the variety of methods used in routine practice in the participating centres. We also aimed at testing sample types and parasite concentrations close to low concentrations routinely found in human samples in this condition [28]. The lack of consistency of certain results may be explained by this intrinsic diversity of matrices and PCR assays. In this respect, it was interesting to calculate the final parasite amounts to be detected per PCR reaction in each sample type depending on the methods used (Table 1). Indeed, even though the starting concentrations in the samples (500, 200 and 20 Tg/mL) were standardized, the diversity of DNA extraction and PCR methods lead to a great heterogeneity in the input of Tg in the PCR reaction among the six centres. Logically, when this number was very low (≤1 Tg/PCR: 0.13 Tg/PCR and 1 Tg/PCR in CSF and ’small WB + PRU’, respectively), the PCR performances significantly decreased, or the Cp mean increased, with storage duration. Similarly, for other sample types (’large WB+RH’ or BC), the low *Toxoplasma* DNA input (4 Tg/reaction) likely corresponds to the detection threshold in these experimental conditions, since the performance scores were below 100% throughout the storage duration (Table 2).

The effects of storage temperature and duration have been previously evaluated on different sample types, for example on blood samples to isolate human DNA and RNA [29, 30]. However the objectives of these recent studies were mainly focused on the preservation of biological material in the context of biobank practices. Two reports specifically studied the impact of storage conditions on the sensitivity of diagnostic tools for toxoplasmosis [31, 32]. James *et al*. showed that the PCR detection of *Toxoplasma* parasites resuspended in water was reduced after storage at +4°C for 48 h [31]. Closer to routine practice, Joss *et al*. used spiked AF with tachyzoites, but the high number of false positive results in that work using nested PCR casts doubts on their conclusions [32].

As compared with previous studies, the use in our study of several types of matrices, whether paucicellular matrices such as AF, BALF, CSF, or cell-rich matrices such as WB and BC, is both original and interesting. We evaluated the stability of different samples obtained from blood, because, even though BC appears as the best sample for the diagnosis of disseminated toxoplasmosis [22], WB is used by many clinical microbiology laboratories. Additionally, depending on local practices, BC can be collected immediately before shipment or at reception in the reference laboratory, which is why we investigated both BC storage and BC after WB storage. This is the first time that so many different *Toxoplasma-*spiked matrices are studied.

In conclusion, our work allows proposing guidelines for good laboratory practices, which are required for microbiological diagnosis to follow Quality Management Systems. Provided that real-time PCR is performed in duplicate, the storage of samples used to diagnose toxoplasmosis is possible at +4°C for up to 7 days. However, a note of caution is in order for CSF and WB samples, which, if they are stored for more than 4 days at +4°C before analysis, should be tested at least in triplicate to maintain the sensitivity of molecular diagnosis on biological samples with low parasitic loads.

## Supporting information

S1 Table PCR performance scores and mean of Cp values for different artificially spiked-samples without and after storage at +4°C during 2, 4 and 7 days before processing for molecular diagnosis of toxoplasmosis: Complete results. (DOCX)
