# Supplementary material for: Impact of pre-analytic step duration on molecular diagnosis of toxoplasmosis for five types of biological samples
Source: PLoS One. 2021 Feb 17;16(2):e0246802. doi: 10.1371/journal.pone.0246802 (PMC7888589; doi:10.1371/journal.pone.0246802)
Supplement: S1 Table — (DOCX) [file pone.0246802.s001.docx]

**S1 Table. PCR performance scores and mean of Cp values for different artificially spiked-samples without and after storage at +4°C during 2, 4 and 7 days before processing for molecular diagnosis of toxoplasmosis : complete results.**

| Sample type  (No. of samples extracted) | Inoculum  Tg/mL |  | Without storage  (D0) | After 2 days (D2) | After 4 days (D4) | After 7 days (D7) |
| --- | --- | --- | --- | --- | --- | --- |
| AF + RH | 500 | No. of PCR+ ^a^ | 9/9 | 9/9 | 9/9 | 9/9 |
| (n=37) |  | Mean Cp ±SD ^b^ | 23.45±0.24 | 23.41±0.1 | 23.26±0.11 | 23.40±0.11 |
|  | 100 | PCR+ | 9/9 | 9/9 | 9/9 | 9/9 |
|  |  | Cp +/-SD | 25.65+/-0.13 | 25.69+/-0.2 | 25.79+/-0.16 | 25.73+/-0.07 |
|  | 20 | PCR+ | 9/9 | 9/9 | 9/9 | 9/9 |
|  |  | Cp +/-SD | 27.82+/-0.6 | 27.83+/-0.47 | 28.28+/-0.34 | 28.30+/-0.25 |
| CSF + RH | 500 | PCR+ | 6/6 | 6/6 | 6/6 | 6/6 |
| (n=37) |  | Cp +/-SD | 28.33+/-1.09 | 28.10+/-0.72 | 28.24+/-0.85 | 28.71+/-0.67 |
|  | 100 | PCR+ | 6/6 | 6/6 | 6/6 | 6/6 |
|  |  | Cp ±SD | 32.03±1.84 | 34.23±1.73 | 33.98±1.38 | 32.04±1.73 |
|  | 20 | PCR+ | 12/12 | 11/12 | 10/12 | 6/12* |
|  |  | Cp ±SD | 33.81±2.06 | [34.50±1.42] | [34.50±1.77] | [33.69±1.77] |
| BALF + RH | 1000 | PCR+ | 6/6 | 6/6 | 6/6 | 6/6 |
| (n=37) |  | Cp ±SD | 29.18±0.29 | 28.82±0.27 | 29.29±0.32 | 29.03±0.19 |
|  | 200 | PCR+ | 6/6 | 6/6 | 6/6 | 6/6 |
|  |  | Cp ±SD | 31.88±0.46 | 31.60±0.23 | 31.50±0.84 | 30.84±0.10 |
|  | 40 | PCR+ | 9/9 | 9/9 | 9/9 | 9/9 |
|  |  | Cp ±SD | 34.23±0.31 | 34.67±0.94 | 33.47±0.42 | 33.75±0.23 |
| Small WB + PRU | 500 | PCR+ | 9/9 | 9/9 | 9/9 | 9/9 |
| (n=37) |  | Cp ±SD | 26.32±1.22 | 25.89±0.64 | 28.25±0.90 | 26.68±0.72 |
|  | 100 | PCR+ | 9/9 | 9/9 | 9/9 | 9/9 |
|  |  | Cp ±SD | 28.66±0.80 | 28.49±0.48 | 30.64±0.75* | 29.11±1.06 |
|  | 20 | PCR+ | 9/9 | 9/9 | 9/9 | 9/9 |
|  |  | Cp ±SD | 30.90±0.58 | 32.18±0.86* | 32.64±1.16* | 32.95±1.96* |
| Large WB + RH, | 500 | PCR+ | 6/6 | 6/6 | 6/6 | 4/6 (NS) |
| and BC after storage |  | Cp ±SD | 30.31±0.94 | 27.16±0.44 | 27.52±0.60 | [33.65±5.09] |
| (n=37) | 100 | PCR+ | 6/6 | 6/6 | 6/6 | 3/6 (NS) |
|  |  | Cp±SD | 33.22±1.93 | 30.85±0.85 | 30.48±0.81 | [34.84±3.28] |
|  | 20 | PCR+ | 3/6 | 5/6 | 5/6 | 2/6 |
|  |  | Cp ±SD | [34.58±2.86] | [33.93±0.97] | [33.37±2.66] | [35.79±3.52] |
| Large WB + | 500 | PCR+ | 6/6 | 6/6 | 6/6 | 6/6 |
| infected-THP1 and |  | Cp ±SD | 23.38±0.45 | 26.73±0.30 | 23.84±0.17 | 22.99±0.32 |
| BC after storage | 100 | PCR+ | 6/6 | 2/6 ^c^ | 6/6 | 6/6 |
| (n=37) |  | Cp ±SD | 26.26±0.85 | [30.10±1.59] | 26.03±0.48 | 25.08±0.49 |
|  | 20 | PCR+ | 6/6 | 3/6^c^ | 6/6 | 6/6 |
|  |  | Cp ±SD | 27.80±0.46 | [35.41±4.40] | 28.06±0.48 | 27.92±1.04 |
| BC + RH | 500 | PCR+ | 9/9 | 9/9 | 9/9 | 9/9 |
| (n=37) |  | Cp ±SD | 30.48±0.65 | 30.47±1.15 | 30.99±0.85 | 30.03±0.64 |
|  | 100 | PCR+ | 9/9 | 9/9 | 9/9 | 9/9 |
|  |  | Cp ±SD | 34.74±0.81 | 34.23±0.86 | 35.45±1.02 | 33.96±0.7 |
|  | 20 | PCR+ | 6/9 | 8/9 | 8/9 | 8/9 |
|  |  | Cp ±SD | [36.68±0.76] | [34.95±1.92] | [36.56±2.05] | [35.38±1.12] |

For each storage duration at + 4°C and *T. gondii* concentration, three different samples were extracted in parallel and at least 2 PCR were performed for each sample.

Tg : *Toxoplasma gondii*; Cp : crossing point; SD : standard deviation; AF : amniotic fluid; CSF : cerebrospinal fluid; BALF : bronchoalveolar lavage fluid; WB : whole blood; BC : buffy coat; RH : RH *Toxoplasma* strain; PRU : Prugniaud *Toxoplasma* strain ; NS: number of positive PCR at D7 not statistically significant compared to number of positive PCR without conservation (D0)

^a^: number of positive PCR/ number of performed PCR

^b^: mean of Cp ±SD

^c^ : a technical problem during the buffy coat isolation may explain these two discrepant data (see the Discussion section)

* p<0.05 compared with Cp measured in samples without conservation
